# Yttrium-Modified B_12_N_12_ Nanocages for High-Performance H_2_ Sensing: Insights from DFT Calculations on Sensitivity, Selectivity, and Recovery

**DOI:** 10.1021/acsomega.5c11273

**Published:** 2026-01-21

**Authors:** Wellington da Conceição Lobato do Nascimento, Natanael de Sousa Sousa, Adilson Luís Pereira Silva, Adeilton Pereira Maciel

**Affiliations:** † 37892 Universidade Federal do Maranhão, 65080-805 São Luís, MA, Brazil; ‡ 119484 Universidade Estadual do Maranhão, 65055-310 São Luís, MA, Brazil; § 28123 Universidade Federal do Rio Grande do Norte, 59078-970 Natal, RN, Brazil

## Abstract

Boron nitride (B_12_N_12_) nanocages have attracted considerable attention due to their exceptional structural stability and tunable electronic properties, making them promising candidates for gas-sensing applications. In this study, DFT-D3 calculations at the B3LYP/def2-TZVP level, including relativistic effects for yttrium (SARC-ZORA-def2-TZVP), were employed to investigate H_2_ adsorption on pristine and Y-modified (doped, decorated, and encapsulated) B_12_N_12_ nanocages. The pristine nanocage exhibited weak physisorption (*E*_ads_ = −0.04 eV), whereas the Y@b_64_ configuration demonstrated strong chemisorption (*E*_ads_ = −0.96 eV), pronounced electronic sensitivity (Δ*E*_gap_ = 74.94%), and a feasible recovery time (τ = 166.8 s). Analyses of electrostatic potential, molecular dynamics (1000 ps), IR, and UV–vis spectra confirmed the structural robustness and optical detectability of H_2_. Furthermore, the Y@b_64_ nanocage showed remarkable selectivity toward H_2_ compared to common interfering gases (CH_4_, CO, H_2_S, and N_2_). Overall, Y@b_64_ combines high adsorption energy, strong sensitivity, and efficient recovery time, underscoring its potential as a selective, stable, and high-performance H_2_ gas sensor.

## Graphical abstract

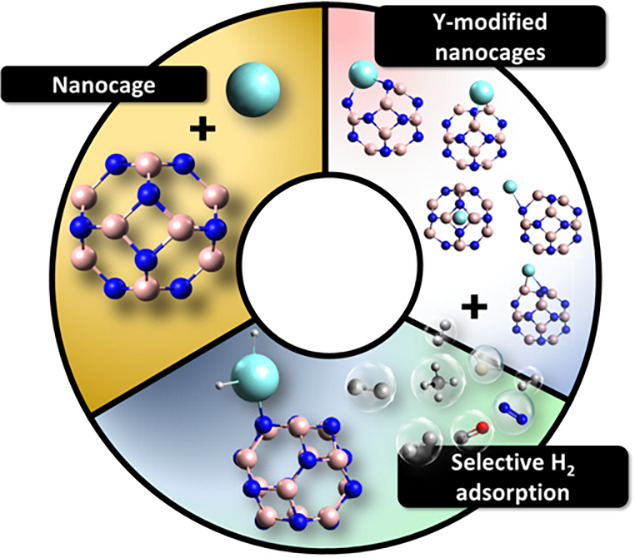

## 1 Introduction

The development of sensors for odorless, colorless, and toxic gases such as H_2_, NH_3_, SO_2_, O_2_, NO_2_, H_2_S, CO, and CO_2_ has generated great interest in the scientific community and industry.^1–3^ Sensors capable of quickly and accurately identifying these gases play a crucial role in mitigating environmental impacts, such as global warming, and in advancing alternative energy sources. In the case of hydrogen (H_2_), efficient sensors are essential to ensure safety and efficiency in industrial processes such as coal mining, nuclear reactors, and semiconductor manufacturing, where rigorous monitoring is required to reduce risks and optimize operations.^4–7^

Nanomaterials, including nanosheets, nanotubes, and nanocages, have been widely studied as gas sensors due to their exceptional electronic properties and high surface area.^8^ Among them, boron nitride-based structures stand out, such as nanotubes (BNNTs), two-dimensional structures (h-BN), nanoclusters (B_6_N_6_), and nanocages (BN)*_n_*, combining sensitivity, rapid detection, and short recovery times. Theoretical studies using DFT calculations have demonstrated the efficiency of these systems in the adsorption of various gases. For example: Aasi et al.^9^ observed that Pt- and Pd-decorated novel green phosphorene exhibit high reactivity for C_2_H_2_, H_2_, and CH_4_; Kalateh et al.^10^ showed that B_16_N_16_ has superior electronic adsorption properties for H_2_ compared to C_32_ and B_8_C_24_; and Choir et al.^11^ found that B_12_N_12_ nanocages interact spontaneously and exothermically with CO_2_ and H_2_ (*E*_ads_ = −26.86 and −6.94 kJ/mol, respectively). Nair et al.^12^ also demonstrated that B_12_N_12_ strongly adsorbs gases such as AsH_3_, H_2_Se and H_2_S, while H_2_ and CH_4_ present weaker interactions, with relevant modifications in reactivity indices, such as chemical potential and hardness.

Despite the potential of B_12_N_12_ for atmospheric gas sensors, some molecules such as H_2_, H_2_S, CO, COCl_2_, and NO do not spontaneously adsorb on the pure cage due to weak interactions, requiring structural modifications to optimize their electronic properties.^13–17^ The insertion of transition metals is an efficient strategy to increase the affinity of the nanocage for different gases. Experimentally, Oku et al.^18–21^ synthesized B_12_N_12_ and B_36_N_36_ cages, pure and modified with transition metals (Fe, Ag, La, and Y), confirming their structures by electron microscopy and mass spectrometry. Yttrium, in particular, proved effective in improving H_2_ adsorption, for example: (i) Wang and Tian^22^ indicated, via DFT, that each Y atom in a C_48_B_12_ cage can bind up to six H_2_ molecules with significant binding energies, suggesting chemisorption-type interactions; (ii) Kundu and Chakraborty^23^ showed, via DFT, that Y atom attached on Triazine can adsorb seven H_2_ molecules with binding energy of 0.33 eV/H_2_ leading to 7.3 wt % of H_2_; (iii) Srivastava et al.^24^ reported experimentally that the chemoresistive gas sensor based on Y-CeO exhibited excellent long-term stability, outstanding sensitivity, fast response and recovery times, and selectivity to H_2_ in comparison to other gases. Revealing the exceptional potential of the Y-CeO sensor as a low-trace H_2_ gas detector; (iv) Ferlazzo et al.^25^ showed experimentally that the novel yttria-doped ZrO_2_ exhibited excellent characteristics in terms of sensor response, fast response and recovery time (5 and 10 s, respectively) and good selectivity to H_2_.

Therefore, all of these findings motivated us to design a system that efficiently binds the H_2_ gas by structural modification of B_12_N_12_ nanocage with Y metal and helps in detecting this gas in the environment. The novelty of this work lies in the comprehensive comparative analysis of five distinct yttrium-based configurations, offering a deeper understanding of the electronic and structural factors that govern the interaction between H_2_ molecules and the nanocage surface.

This study aims to fill this gap by employing density functional theory (DFT) calculations, including relativistic effects, to elucidate how different yttrium insertion strategies influence the adsorption behavior of H_2_. By mapping these effects, we identify key mechanisms that enhance selectivity, sensitivity, and adsorption efficiency, ultimately contributing to the rational design of high-performance hydrogen sensors. In this context, our investigation aims to identify a material with the potential to be applied as a selective sensor, exhibiting high sensitivity and rapid detection of hydrogen gas in the environment.

## 2 Computational Methodology

Density Functional Theory (DFT) calculations were performed using ORCA 5.0.^26^ The isolated and yttrium-modified B_12_N_12_ nanocages were optimized with the B3LYP functional and Grimme D3 dispersion correction,^27,28^ suitable for long-range interactions. The B3LYP-D3 functional was adopted due to its accuracy in geometries and vibrational frequencies, combined with low computational cost. Thus, B3LYP stands out as a stable, economical and reproducible option, as validated in gas adsorption studies.^29,30^ Different basis sets were used: def2 with relativistic effects SARC-ZORA-def2-TZVP for yttrium and ZORA-def2-TZVP for the other elements. The adopted convergence criteria were: RMS gradient (5 × 10^–6^ Hartree), RMS shift (1 × 10^–4^ Hartree/Bohr), maximum gradient (2 × 10^–3^ Bohr), maximum shift (3 × 10^–4^ Hartree/Bohr) and energy (4 × 10^–3^ Hartree). Frequency analyses confirmed the global minima of the optimized structures. The structural modification of B_12_N_12_ with yttrium gave rise to five distinct structures:^13,31^

- Y-doped (YB_11_N_12_), in which one B atom of B_12_N_12_ is replaced by a Y atom;
- Y-doped (YB_12_N_11_), in which one N atom of B_12_N_12_ is replaced by a Y atom;
- Y-decorated (Y@b_64_), in which a Y atom is externally adsorbed onto the B_12_N_12_ nanocage, positioned above one b_64_ bond that connects a hexagonal and a tetragonal ring;
- Y-decorated (Y@b_66_), in which a Y atom is externally adsorbed onto the B_12_N_12_ nanocage, positioned above one b_66_ bond that connects two hexagonal rings;
- Y-encapsulated (Y@B_12_N_12_), where a Y is located inside the B_12_N_12_ nanocage.

The nanocages were optimized in their neutral forms (charge = 0). Preliminary spin-state tests showed that B_12_N_12_, YB_11_N_12_, and B_12_N_11_Y converge to closed-shell singlet states (multiplicity = 1), confirming their diamagnetic character (see Table S1 in Supporting Information). In contrast, the Y-decorated and -encapsulated structures (Y@b_64_, Y@b_66_, and Y@B_12_N_12_) exhibit open-shell doublet ground states (multiplicity = 2), as evidenced by the distinct α/β orbital energies (Tables 1 and 3) and a total spin population of 1.0, indicating the presence of one unpaired electron. After H_2_ adsorption, all complexes retain the multiplicity of their corresponding ground states, showing that the magnetic behavior induced by yttrium remains unchanged upon interaction with the adsorbates. The stability of the systems was evaluated by determining the cohesive energy (*E*_coh_) following eq 1 ^32^

1 $$E_{\mathrm{coh}}=\frac{1}{N}\left(E_{\mathrm{nanocage}}-xE_{B}-yE_{N}-zE_{Y}\right)$$

in this equation, *E*_nanocage_ represents the total energy of the nanocage, whether pure or modified with yttrium. *E*_B_, *E*_N_, and *E*_Y_ correspond to the energies of the boron, nitrogen, and yttrium atoms. The variables *x*, *y*, and *z* denote the number of B, N, and Y atoms in the structure, while N represents the total number of atoms of the studied nanocages.

**Table 1:** Calculated Values of Formation Energy (*E*_form_), Cohesive Energy (*E*_coh_), Dipole Moment (DM), NPA Charge on the Y Atom (*Q*_Metal_), Energy Gap (*E*_gap_) and Work Function (Φ) for Isolated Systems

system	*E*_form_ (eV)	*E*_coh_ (eV)	DM (Debye)	*Q*_Metal_ (|e|)	*E*_gap_ (eV)	Φ (eV)
B_12_N_12_	–3.04	–7.33	0.00		6.80	4.46
YB_11_N_12_	–2.96	–7.25	9.75	2.024	3.72	4.70
B_12_N_11_Y	–2.91	–6.93	8.89	1.310	2.54	3.83
Y@b_64_	–2.92	–7.07	5.90	1.134	2.43^a^	3.14
					2.93^b^	
Y@b_66_	–2.91	–6.99	5.64	0.986	2.30^a^	3.72
					1.64^b^	
Y@B_12_N_12_	–2.58	–6.71	1.79	0.576	2.07^a^	3.41
					2.33^b^	

a Spin up.

b Spin down.

For thermodynamic analysis of the most viable systems to be synthesized and to compare with the cohesion energy data, we also calculated the formation energy of the nanocages, according to eq 2 ^33^

2 $$E_{\mathrm{form}}=\frac{\left(E_{\mathrm{modified}}-a{\mathrm{E̅}}_{B}-b{\mathrm{E̅}}_{N}-c{\mathrm{E̅}}_{Y}\right)}{N}$$

where the *E*_modified_ is the total energy nanocage modified, *E̅*_B_, *E̅*_N_ and *E̅*_Y_ are the energies for elements in B_2_, N_2_ and cluster of the Y atom, respectively; *a*, *b*, and *c* are the numbers of B, N, and Y atoms, respectively, and *N* is the total number of atoms.

The energy gap *E*_gap_ is defined as the difference between the energy levels LUMO (*E*_LUMO_) and HOMO (*E*_HOMO_). The electronic sensitivity (Δ*E*_gap_) of the interaction between the H_2_/nanocages was determined using eq 2.^14^

3 $$\Delta E_{\mathrm{gap}}=\left[\frac{(E_{\mathrm{gap}\left(\mathrm{nanocage}‐H_{2}\right)}-E_{\mathrm{gap}\left(\mathrm{nanocage}\right)})}{E_{\mathrm{gap}\left(\mathrm{nanocage}\right)}}\right]\times 100$$

the *E*_gap(nanocage‑H_2_)_ represents the energy gap associated with the interaction of H_2_ with both pure and modified B_12_N_12_ nanocages, while *E*_gap(nanocage)_ refers to the energy gap of pure or Y-modified B_12_N_12_. To better understand the spontaneity of the cage/gas adsorption process, thermodynamic parameters were analyzed. Specifically, the variation in adsorption enthalpy (Δ*H*_ads_) and free energy (Δ*G*_ads_) using eqs 4 and 5.^11^

4 $${\Delta G}_{\mathrm{ads}}=G_{\mathrm{gas}‐\mathrm{nanocage}}-\left(G_{\mathrm{gas}}+G_{\mathrm{nanocage}}\right)$$

5 $${\Delta H}_{\mathrm{ads}}=H_{\mathrm{gas}‐\mathrm{nanocage}}-\left(H_{\mathrm{gas}}+H_{\mathrm{nanocage}}\right)$$

*G*_gas‑nanocage_ and *H*_gas‑nanocage_ represent the free energy and enthalpy of gas-nanocage adsorption. *H*_gas_ and *G*_gas_ are related to the energies of H_2_ gas and *G*_nanocage_ and *H*_nanocage_ are the energies of the isolated nanocages, respectively. Equation 5 was used to obtain adsorption energy (*E*_ads_) values to study the interaction between B_12_N_12_ or yttrium-modified nanocages and H_2_ gas.^16^

6 $$E_{\mathrm{ads}}=E_{\left(\mathrm{nanocage}‐H_{2}\right)}-\left(E_{\left(\mathrm{nanocage}\right)}+E_{\left(H_{2}\right)}\right)+E_{\mathrm{BSSE}}$$

the system energy *E*_(nanocage‑H_2_)_ corresponds to the B_12_N_12_ or Y–B_12_N_12_ nanocage with H_2_, including the zero-point vibrational energy (ZPVE), while *E*_(nanocage)_ and *E*_(H_2_)_ refer to the energies of the isolated nanocages and the H_2_ molecule. The error correction of the basis set superposition (BSSE) was applied for greater accuracy in the interactions. The recovery time (τ) is given by eq 7 ^34–36^

7 $$\tau =v_{0}^{-1}e^{-E_{\mathrm{ads}}/k_{B}T}$$

with v_0_ = 10^12^ s^–1^, *k*_B_ = 8.62 × 10^–5^ eV K^–1^ and *T* is the thermodynamic temperature (K).^37^ *E*_ads_ values between −0.3 and −1.0 eV result in τ of order of seconds,^38^ indicating a sensor potential. The work function (Φ) was calculated from the frontier orbitals, and the variation (ΔΦ) before and after adsorption was the criterion for evaluating the applicability of the nanocages (eqs 8 and 9).

8 $$\Phi \approx E_{H}+\frac{1}{2}\left(E_{L}-E_{H}\right)$$

9 $$\Delta \Phi =|\frac{\left(\Phi_{\left(\mathrm{nanocage}‐H_{2}\right)}-\Phi_{\left(\mathrm{nanocage}\right)}\right)}{\Phi_{\left(\mathrm{nanocage}\right)}}\times 100|$$

where Φ_(nanocage–H_2_)_ represents the work function values of the adsorbed systems (B_12_N_12_/H_2_) and Φ_(nanocage)_ corresponds to the work function of the pure B_12_N_12_ or Y-modified nanocage. The quantum descriptor parameters were obtained based on the values of frontier orbitals (HOMO and LUMO)^39,40^ by applying eqs 10–15.

10 $$\mathrm{IP}\approx {-E}_{\mathrm{HOMO}}$$

11 $$\mathrm{eA}\approx {-E}_{\mathrm{LUMO}}$$

12 $$\eta \approx \frac{1}{2}\left(E_{\mathrm{LUMO}}-E_{\mathrm{HOMO}}\right)$$

13 $$\mu \approx \frac{1}{2}\left(E_{\mathrm{LUMO}}+E_{\mathrm{HOMO}}\right)$$

14 $$S\approx \frac{1}{2\eta }$$

15 $$\omega =\frac{\mu^{2}}{2\eta }$$

The electrostatic potential map (MEP) and density of state spectra (DOS) were obtained using the MultiWfn program,^41^ in addition to UV–vis spectra, in order to investigate the electronic and optical properties of the cage/gas interactions, elucidating the electronic distribution, reactivity and sensing potential toward H_2_. The stability of the most favorable system after H_2_ adsorption was evaluated by quantum molecular dynamics (MD) of 1000 ps, with an integration interval of 2 fs, using the GFN-1 Hamiltonian in the xTB software.^42^

The electrical conductivity (σ) can be employed to investigate the interactions of pure and modified B_12_N_12_ nanocages with H_2_. Its value was calculated as eq 16 ^43,44^

16 $$\sigma ={\mathrm{AT}}^{3/2}e^{\left(-E_{\mathrm{gap}}/2k_{B}T\right)}$$

where *A* is a constant (electron m^–3^ K^–3/2^), *E*_gap_ is the energy gap, *k*_B_ is the Boltzmann constant (8.62 × 10^–5^ eV K^–1^) and *T* is the thermodynamic temperature (K). Based on this, the most promising yttrium-doped nanocage for H_2_ detection was subjected to interactions with interfering gases (CH_4_, CO, H_2_S and N_2_), with selectivity being analyzed by calculating the sensor response (*S*) and the selectivity coefficient (κ), according to eqs 17 and 18.^45,46^

17 $$S=\frac{\left|R_{\mathrm{gas}}-R_{\mathrm{pure}}\right|}{R_{\mathrm{pure}}}=\frac{\left|\frac{1}{\sigma_{\mathrm{gas}}}-\frac{1}{\sigma_{\mathrm{pure}}}\right|}{\frac{1}{\sigma_{\mathrm{pure}}}}=\frac{\left|\sigma_{\mathrm{pure}}-\sigma_{\mathrm{gas}}\right|}{\sigma_{\mathrm{gas}}}$$

18 $$\kappa_{H_{2}\text{−}\mathrm{int}}=\frac{S_{H_{2}}}{S_{\mathrm{int}}}$$

the σ_gas_ represents the conductivity of the gas adsorbed, σ_pure_ is the conductance of the isolated nanocage and *R*_gas_ and *R*_pure_ represent the electrical resistance of the gas/cage system and pure nanocage, respectively. In eq 18, *S*_H_2__ and *S*_int_ indicate sensitivity to H_2_ gas and to interferents gases, respectively, and κ_H_2_–int_ represents the sensitivity ratio to H_2_ against an interfering gas.

## 3 Results and Discussion

### 3.1 Structural Analysis

The structure of the B_12_N_12_ nanocage was optimized, presenting a configuration with eight hexagonal and six tetragonal rings (Figure 1). In this geometry, all B and N vertices are equivalent. Two types of B–N bonds were identified: b_64_, between hexagonal and tetragonal rings, with a length of 1.435 Å, and *b*_66_, between hexagonal rings, with a length of 1.483 Å. These values are close to those reported by Zhao et al.,^47^ who studied the structural and electronic properties of B_12_N_12_ doped with transition metals. For the tetragonal rings, the B–N–B and N–B–N angles were 80.5° and 98.9°, respectively, in agreement with the literature.^48–51^

**Figure 1:**
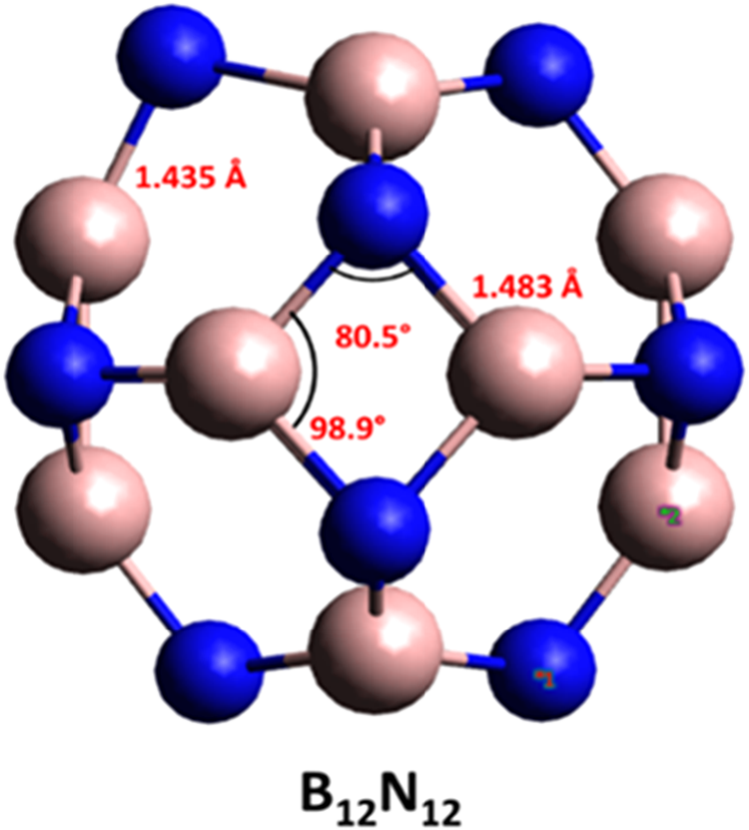
Optimized structure of B_12_N_12_ nanocage with B3LYP functional and ZORA-def2-TZVP basis set.

The optimized structures of the pristine and yttrium-modified B_12_N_12_ nanocages (YB_11_N_12_, B_12_N_11_Y, Y@b_64_, Y@b_66_, and Y@B_12_N_12_) are shown in Figure 2. The replacement of B or N atoms by yttrium causes evident structural deformations, attributed to the larger atomic radius of Y compared to B and N. In the decorated structures, Y@b_64_ and Y@b_66_, the B–N bond lengths near the metal increased to 2.460 Å and 2.380 Å, respectively, indicating significant local distortions. Similarly, the encapsulation in Y@B_12_N_12_ resulted in bond lengths of 2.620 Å (*b*_66_) and 2.360 Å (*b*_64_), greater than those of the pristine structure. These results demonstrate that yttrium modification, whether through doping, decoration, or encapsulation, induces structural changes in the B_12_N_12_ nanocage. The expansion of B–N bonds, in turn, directly affects electronic stability and molecular adsorption, aspects detailed in subsequent analyses. However, despite the structural changes, it is clear that all yttrium-modified structures are stable over 1000 ps, as shown by the molecular dynamics results.

**Figure 2:**
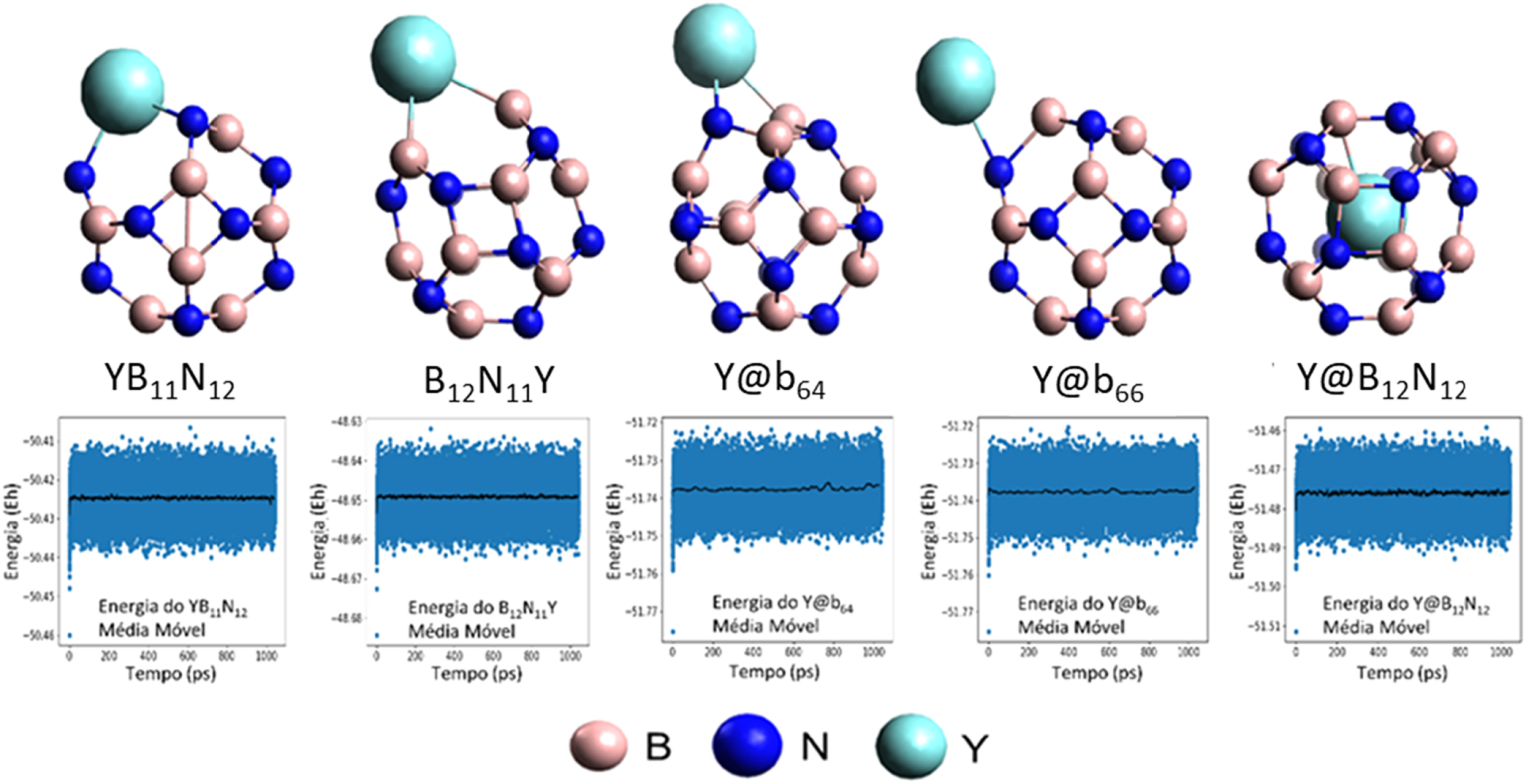
Illustration of the optimized structures and molecular dynamics of the B_12_N_12_ nanocage and the yttrium metal-modified nanocages: doped (YB_11_N_12_ and B_12_N_11_Y), decorated (Y@b_64_ and Y@b_66_), and encapsulated (Y@B_12_N_12_).

In Table 1, we present the formation energy (*E*_form_), cohesive energy (*E*_coh_), dipole moment (DM), NPA charge on the Y atom (*Q*_Metal_), energy gap (*E*_gap_), and work function (Φ) for the pristine B_12_N_12_ and its yttrium-modified nanocages. The α-spin (spin up) and β-spin (spin down) orbital energies are reported for the open-shell systems. The pristine nanocage exhibited zero DM, while YB_11_N_12_ presented the highest value (9.75 D), and Y@B_12_N_12_ the lowest (1.79 D), reflecting a lower charge partitioning in the encapsulation.^52,53^ These results are corroborated by the lower charges on the yttrium atom. The *E*_coh_ analysis indicates that the doped, decorated or encapsulated structures are less stable than the pristine B_12_N_12_, although YB_11_N_12_ stands out as the most stable among them (*E*_coh_ = −7.25 eV). In addition, the negative formation energy for all the nanocages confirms their thermodynamic stability.^54,55^

In this study, it was employed to investigate the interaction between the transition metal yttrium (Y) and the B_12_N_12_ nanocage, as presented in Table 1. The natural charges (NPA) indicate that Y acts as an electron donor, transferring electron density to the nanocage due to its lower electronegativity. Among the evaluated structures, the YB_11_N_12_-doped nanocage exhibited the highest charge transfer, demonstrating that substituting a boron atom with Y enhances electronic redistribution and directly influences the electronic and chemical properties of the nanocage. The work function (Φ) values, calculated by eq 8, were reduced after the modification with yttrium, except in YB_11_N_12_ (Φ = 4.70 eV), which maintained the highest value. The decorated system Y@b_64_ presented the lowest (Φ = 3.14 eV), evidencing the impact of the decoration on the electronic response.

### 3.2 Adsorption Analysis

Figure 3 shows the structures resulting from H_2_ adsorption on the nanocages. Before locating the minimum-energy complexes, we explored different initial adsorption orientations. For the B_12_N_12_ and Y@B_12_N_12_ systems, we tested positions over B and N atoms. In the doped and decorated nanocages, H_2_ was initially placed near the metal, allowing full geometric relaxation to reach the most stable configuration. Only a single initial position of H_2_ was required for each system, as hydrogen is a small molecule that adjusts spontaneously during optimization. In the doped B_12_N_11_Y–H_2_ and decorated Y@b_66_–H_2_ systems, H–H bond rupture and formation of new H–B and H–Y bonds were observed. In the Y@b_64_–H_2_ system, the H_2_ molecule dissociated and bonded to the yttrium atom, giving rise to the H–Y–H configuration. This behavior was also reported by Ahangari and Mashhadzadeh,^56^ verifying H–H bond rupture and formation of new bonds with the cage atoms. For the encapsulated system Y@B_12_N_12_–H_2_, structural deformation occurred, with displacement of the yttrium atom from the center to the outside of the cage. The thermodynamic properties Δ*G*_ads_ and Δ*H*_ads_ (298.15 K), adsorption energy (*E*_ads_), and dipole moment (DM) are listed in Table 2. The B_12_N_12_–H_2_ system showed positive Δ*G*_ads_ (+0.25 eV), characterizing nonspontaneous adsorption. After modification, all systems presented negative Δ*G*_ads_, evidencing spontaneous adsorption. The Δ*H*_ads_ values were predominantly negative, indicating exothermic processes associated with intermolecular interactions, which reduce the energy and increase the stability of the system.

**Figure 3:**
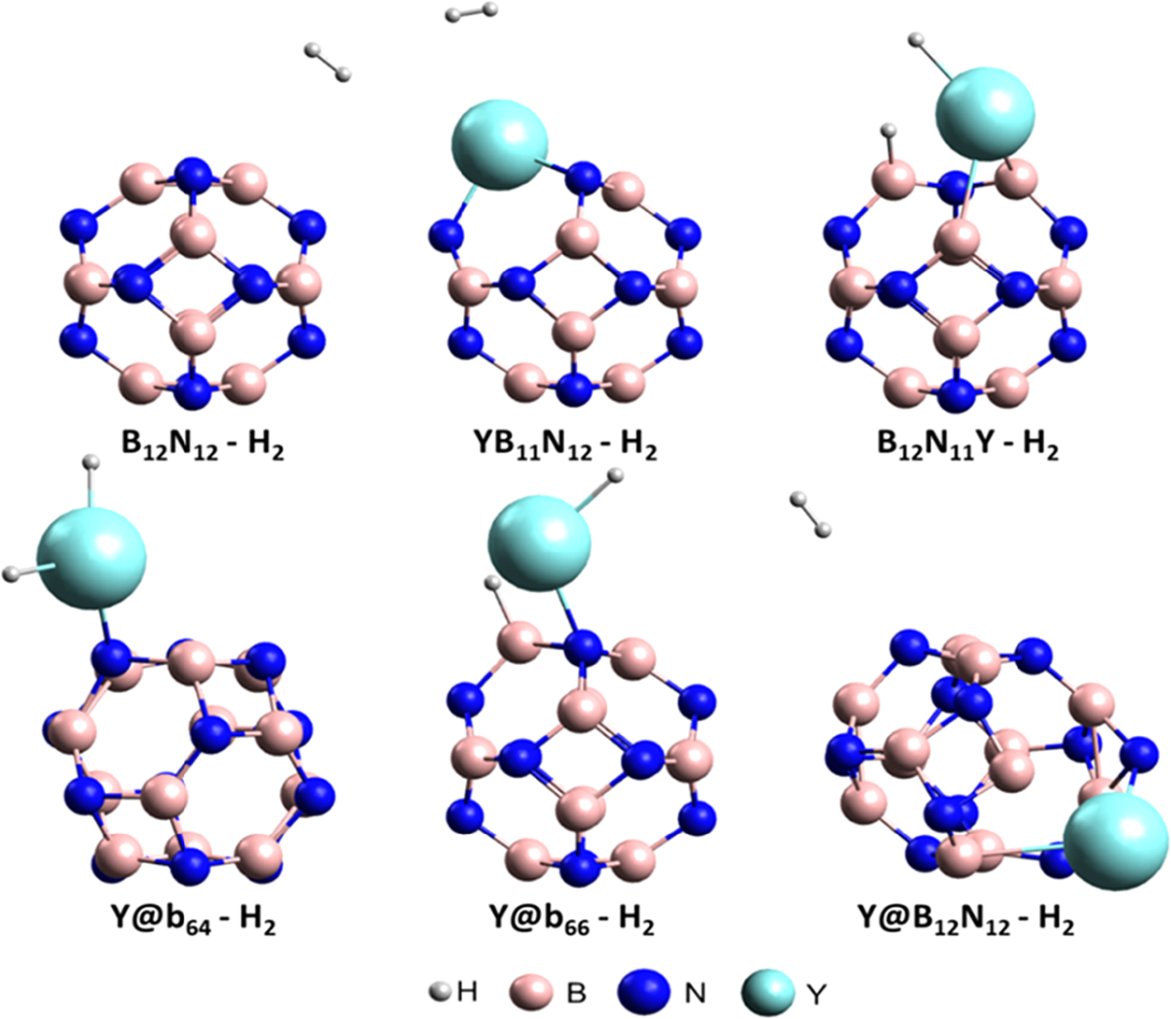
Optimized structures of hydrogen gas (H_2_) adsorption on the surfaces of pure and Y-modified B_12_N_12_ nanocages.

**Table 2:** Values of Gibbs Energy (Δ*G*_ads_), Enthalpy of Adsorption (Δ*H*_ads_), Adsorption Energy (*E*_ads_), Dipole Moment (DM) and Stretching Frequencies H–H (*v*_H_2__) of Pure and Modified B_12_N_12_ after Adsorption with H_2_ Gas

system	Δ*G*_ads_ (eV)	Δ*H*_ads_ (eV)	*E*_ads_ (eV)	DM (Debye)	*v*_H_2__ (cm^–1^)
B_12_N_12_–H_2_	+0.25	0.00	–0.04	0.16	4374.21
YB_11_N_12_–H_2_	–0.16	–0.08	–0.15	10.53	4327.23
B_12_N_11_Y–H_2_	–1.23	–1.61	–2.04	5.94	
Y@b_64_–H_2_	–0.40	–0.76	–0.96	0.83	
Y@b_66_–H_2_	–0.24	–1.85	–2.17	2.54	
Y@B_12_N_12_–H_2_	–7.86	–8.03	–13.24	8.21	4371.54

The adsorption energy (*E*_ads_), obtained by eq 6, indicated physical adsorption for the B_12_N_12_–H_2_ system (−0.04 eV). The nanocages modified with yttrium presented more negative values. However, the *E*_ads_ values of the B_12_N_11_Y–H_2_, Y@b_66_–H_2_ and Y@B_12_N_12_–H_2_ systems were higher (more negative) than −1.0 eV, thus limiting their application as sensors, as it makes the chemisorption process irreversible.^37,38^ On the other hand, the decorated Y@b_64_–H_2_ system presented an adsorption energy value between – 0.3 eV < *E*_ads_ < −1.0 eV,^37,38^ showing that the interaction between Y@b_64_ and H_2_ gas was a moderate chemisorption and suitable for application in H_2_ detection. Regarding the H–H stretching frequency, a decrease was observed for systems without dissociation compared to the isolated H_2_ molecule (*v*_H_2__ = 4412.76 cm^–1^), which also exhibit the lowest charges on the hydrogen atoms, as will be discussed later.

### 3.3 Electronic Analysis

In Table 3, it is observed that the energies of the HOMO and LUMO orbitals of B_12_N_12_ underwent changes after the interaction with H_2_, resulting in a direct variation of the energy gap (*E*_gap_). The B_12_N_12_ gap presented a slight reduction, from 6.8 to 6.62 eV, corresponding to a variation of 2.67%, indicating low sensitivity and weak interaction with H_2_. These results are corroborated by the low adsorption energy of the system (*E*_ads_ = −0.04 eV), characterizing a physisorption (*E*_ads_ > −0.3 eV) with van der Waals-type interaction.^57^ The variation of the work function (ΔΦ), calculated by eq 8 and presented in Table 3, also indicates low sensitivity for the B_12_N_12_–H_2_ system (ΔΦ = 0.93%). In the yttrium-modified systems, the lowest value was observed in YB_11_N_12_–H_2_ (ΔΦ = 0.21%), while the highest occurred in Y@b_64_–H_2_ (ΔΦ = 36.62%), standing out as the most sensitive and promising system for work function-based sensor applications. These data confirm the high sensitivity between the Y@b_64_ nanocage and the H_2_ molecule.

**Table 3:** Values of HOMO-LUMO gap (*E*_gap_), Sensitivity (Δ*E*_gap_), Variation of the Work Function (ΔΦ), NPA Charge on the Y Atom (*Q*_Metal_) and NPA Charge on the H Atoms (*Q*_H_2__), of the Systems, Calculated for the Interaction of H_2_ Gas with Pure and Modified B_12_N_12_

system	*E*_gap_ (eV)	Δ*E*_gap_ (%)	ΔΦ (%)	*Q*_Metal_ (|e|)	*Q*_H_2__ (|e|)
B_12_N_12_–H_2_	6.62	2.67	0.93	–	0.007
YB_11_N_12_–H_2_	3.85	3.39	0.21	2.006	0.033
B_12_N_11_Y–H_2_	2.60	2.61	2.09	1.606	–0.630
Y@b_64_–H_2_	2.57^a^	5.52^a^	36.62	1.782	–1.147
	5.12^b^	74.94^b^			
Y@b_66_–H_2_	1.46^a^	36.62^a^	26.88	1.350	–0.681
	4.82^b^	193.53^b^			
Y@B_12_N_12_–H_2_	3.43^a^	65.47^a^	30.77	1.711	–0.002
	2.60^b^	11.78^b^			

a Spin up.

b Spin down.

The sensitivity of a system is evaluated by the variation of the band gap (Δ*E*_gap_) before and after gas adsorption (eq 3), being influenced by the modification of the nanocage with yttrium. Pure B_12_N_12_ showed low sensitivity to H_2_ (Δ*E*_gap_ = 2.67%; ΔΦ = 0.93%; *E*_ads_ = −0.04 eV), characterizing physisorption. In the modified systems, the doped B_12_N_11_Y–H_2_ was not very sensitive (Δ*E*_gap_ = 2.61%; ΔΦ = 0.21%), while Y@b_66_–H_2_ (β-spin down), Y@b_64_–H_2_ (β-spin down) and Y@B_12_N_12_–H_2_ (α-spin up) showed significant responses (Δ*E*_gap_ = 193.53%, 74.94% and 65.47%; ΔΦ = 36.62% for Y@b_64_). Considering *E*_ads_ and recovery time, Y@b_66_–H_2_ and Y@B_12_N_12_–H_2_ (*E*_ads_ = −2.17 and −13.24 eV) are suitable for H_2_ storage, as recently demonstrated by Sergio and Sousa for the Y@b_64_ nanocage,^58^ while Y@b_64_–H_2_ (*E*_ads_ = −0.96 eV) combines high sensitivity and moderate adsorption energy, being promising as a sensor. Furthermore, analysis of the NPA charges revealed that part of the charge transfers to the hydrogen atoms (with or without H_2_ dissociation) originates from the nanocage. For example, in the YB_11_N_12_–H_2_ system (undissociated H_2_), the charge variation on the Y atom was −0.018 and that on H_2_ was +0.033, whereas in the Y@b_64_–H_2_ system (dissociated H_2_), the charge variation on the Y atom was +0.648 and that on H_2_ was −1.147. This indicates a contribution from the nanocage to the overall charge-transfer process.

The results presented in Table S1 demonstrate that the magnetism induced by yttrium modification significantly affects H_2_ activation, although it is not a necessary condition for molecular adsorption and dissociation. In the Y@b_64_ and Y@b_66_ decorated systems, the nanocage exhibits open-shell behavior prior to adsorption, with multiplicity 2, ⟨*S* ^2^⟩ ≈ 0.75, μ_ef_ in the range of 1.733–1.744 μ_B_, and substantial spin populations on Y (0.939 and 0.840, respectively). After H_2_ adsorption on the Y@b_64_ nanocage, for example, a pronounced decrease in the metal spin population (0.939 → 0.282) is observed, accompanied by spin redistribution to the nanocage atoms and to the hydrogen atoms, which preferentially adsorb on yttrium (see Figure 3). This electronic reorganization is associated with the stronger adsorption energies observed for the decorated systems (−0.96 and −2.17 eV), reflecting the direct participation of partially occupied Y d orbitals in H_2_ activation through σ­(H–H) donation and back-donation into σ*­(H–H).^59^

On the other hand, H_2_ dissociation does not depend exclusively on magnetism. The B_12_N_11_Y nanocage, although diamagnetic (μ_ef_ = 0, ⟨*S* ^2^⟩ = 0), also promotes H–H bond cleavage, as evidenced by the significant negative NPA charges on hydrogen (−0.53 and – 0.09) and by the decrease in the Y electron population (37.7 → 37.4 e^–^). In this case, the process is predominantly governed by charge transfer and electrostatic stabilization, independently of open-shell states. The encapsulated Y@B_12_N_12_ system exhibits an intermediate behavior: before adsorption, the spin population on Y is nearly negligible (0.008) with μ_ef_ = 2.645 μ_B_, but upon interaction with H_2_, local magnetism is induced, increasing the spin on the metal to 0.17 and on the hydrogen atoms to 0.98, consistent with the absence of H_2_ dissociation on the Y@B_12_N_12_ surface. Overall, these results indicate that magnetism plays a central role in the adsorption process, either enhancing the H_2_-nanocage interaction or being induced by adsorption itself, depending on the doping topology and the initial electronic distribution.^60^

To elucidate the electronic interactions associated with H_2_ adsorption on the surfaces of the pristine and Y-modified B_12_N_12_ nanocages, charge density difference (CDD) calculations were performed for the nanocage-H_2_ systems and the corresponding results are shown in Figure 4. It was observed that the interactions between the B_12_N_11_Y, Y@b_64_, and Y@b_66_ nanocages and the H_2_ molecule were more effective, as confirmed by the substantial NPA charge transfers occurring upon adsorption. In these cases, H_2_ dissociation on the nanocage surfaces was identified; however, the most pronounced charge transfer was found in the Y@b_64_–H_2_ system, primarily because the two hydrogen atoms remained bonded to the yttrium atom, in contrast to what was observed in the B_12_N_11_Y–H_2_ and Y@b_66_–H_2_ systems (see Figure 3). The FMO analysis in Figure 5 highlights the electronic distribution and supports the interpretation of gas-nanocage interactions for Y@b_64_ nanocages with H_2_ gas.

**Figure 4:**
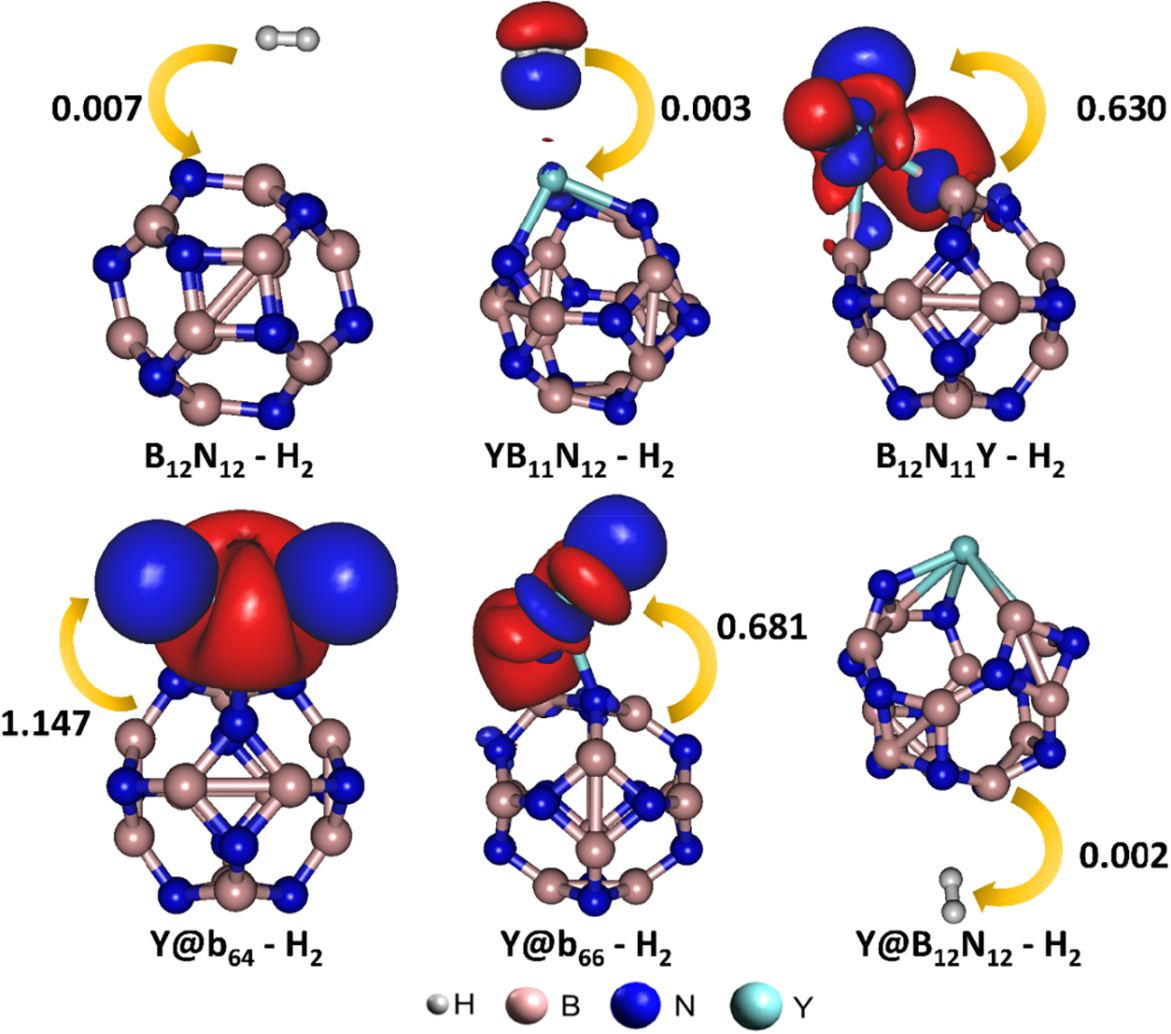
Charge density difference (CDD) of hydrogen gas adsorption on the surfaces of pure and Y-modified B_12_N_12_ nanocages with isosurface of 0.001 e·Å^–3^. Red and blue colors represent charge accumulation and depletion, respectively. The arrows indicate the magnitude and direction of the electronic charge transfer.

**Figure 5:**
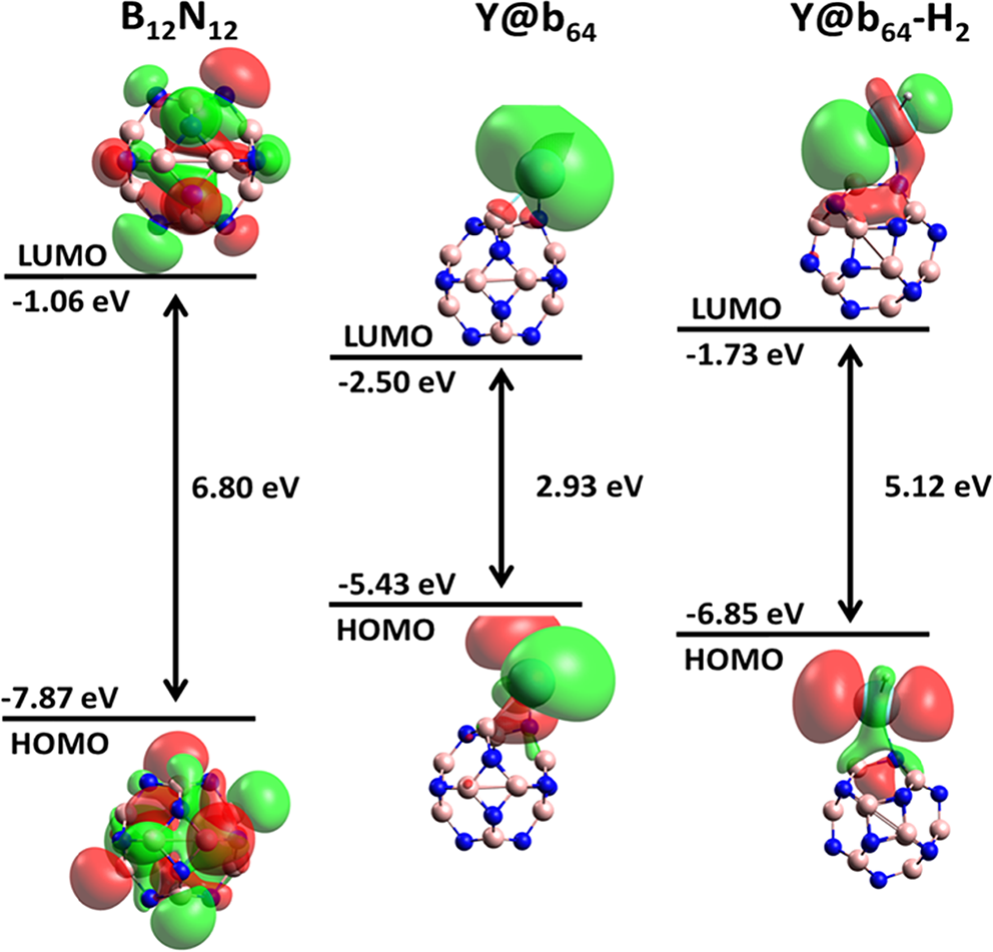
HOMO and LUMO of pure B_12_N_12_ and Y@b_64_ nanocages before and after H_2_ adsorption.

The HOMO presents electron density uniformly distributed over the B and N cage, while the LUMO is slightly concentrated in the peripheral regions of the B_12_N_12_ nanocage. This distribution indicates weak electronic polarization and limited interaction with H_2_, consistent with the observed low adsorption energy (*E*_ads_ = −0.04 eV), small band gap variation (Δ*E*_gap_ = 2.67%), and low work function variation (ΔΦ = 0.93%). In the Y@b_64_ nanocage, the HOMO shows accumulation of electron density near the yttrium atom, suggesting that yttrium acts as an active center for interaction with gases. The LUMO is concentrated in regions close to yttrium and the nanocage, favoring partial charge transfer with H_2_. This explains the higher observed sensitivity (Δ*E*_gap_ = 74.94%) and the moderate adsorption energy (*E*_ads_ = −0.96 eV). After H_2_ adsorption, the HOMO and LUMO orbitals overlap between H_2_ and the doped nanocage, indicating a more significant chemical interaction than in the pure system. The electronic redistribution demonstrates the system’s responsiveness to the presence of H_2_, corroborating the high work function variation (ΔΦ = 36.62%) and the adequate adsorption energy for sensing.

The DOS analysis of both pristine and Y-modified B_12_N_12_ nanocages after hydrogen gas adsorption is presented in Figure S1 in Supporting Information. The DOS plots reveal that upon interaction with H_2_, the B_12_N_12_ nanocage experiences slight HOMO destabilization and LUMO stabilization, leading to a reduction in its energy gap from 6.80 to 6.62 eV. Additionally, all yttrium-modified nanocages exhibited an increase in *E*_gap_ values after H_2_ adsorption compared to the values of the pure nanocages. These findings further support the conclusion that the modified systems are more reactive than the pristine B_12_N_12_ nanocage, as evidenced by the previously discussed parameters.

### 3.4 Quantum Descriptors and Stability

The values of chemical potential (μ), chemical hardness (η), softness (*S*), ionization potential (IP), electron affinity (eA), and electrophilicity (ω) for the isolated systems were calculated by applying eqs 10–15 and are listed in Table 4, as well as for the modified nanocages after gas adsorption. Initially, it is highlighted that the value of the chemical potential is negative in all conditions, implying the stability of the structures and the spontaneity of all processes.^61^ The results show that the pure B_12_N_12_ has the highest hardness (η = 3.38 eV) and the lowest softness (*S* = 0.15 eV^–1^) and electrophilicity (ω = 2.94 eV). These results agree with values published in previous studies.^62–64^ It also shows a higher ionization potential compared to the doped structures, whereas the electron affinity increases upon modification, revealing an enhanced electron-accepting capability. Chemical hardness (η) is associated with a system’s resistance to changes in its electronic distribution;^65^ therefore, larger HOMO–LUMO gaps correspond to more rigid and less reactive systems. Conversely, softness (*S*) characterizes the tendency to accept electronic charge and is inversely related to hardness.^66^ Consequently, systems with smaller HOMO–LUMO gaps become more polarizable and reactive, as observed for Y@B_12_N_12_, whose behavior is consistent with its higher adsorption energy in the Y@B_12_N_12_–H_2_ complex. Among the investigated systems, B_12_N_12_–H_2_ showed the lowest softness (*S* = 0.15 eV^–1^), whereas Y@b_66_–H_2_ exhibited the highest value (*S* = 0.68 eV^–1^). In terms of hardness, Y@b_66_–H_2_ displayed the lowest value (η = 0.73 eV), while B_12_N_12_–H_2_ retained the highest hardness (η = 3.31 eV).^67^ The Y@b_64_–H_2_ system presented intermediate values of chemical potential (μ = −4.29 eV), softness (*S* = 0.20 eV^–1^), and electrophilicity (ω = 3.60 eV).

**Table 4:** Quantum Descriptors: Chemical Hardness (η), Softness (*S*), Ionization Potential (IP), Electron Affinity (eA), Chemical Potential (μ), Electrophilicity (ω)

system	IP (eV)	eA (eV)	η (eV)	μ (eV)	*S* (eV^–1^)	ω (eV)
B_12_N_12_	7.84	1.07	3.38	–4.46	0.15	2.94
B_12_N_12_–H_2_	7.82	1.19	3.31	–4.50	0.15	3.06
YB_11_N_12_	6.73	2.67	2.03	–4.70	0.25	5.44
YB_11_N_12_–H_2_	6.63	2.78	1.92	–4.71	0.26	5.76
B_12_N_11_Y	5.10	2.56	1.27	–3.83	0.39	5.79
B_12_N_11_Y–H_2_	5.05	2.45	1.30	–3.75	0.38	5.42
Y@b_64_	4.35	1.92	1.22	–3.14	0.41	4.04
Y@b_64_–H_2_	6.85	1.73	2.56	–4.29	0.20	3.60
Y@b_66_	4.40	1.97	1.22	–3.18	0.41	4.16
Y@b_66_–H_2_	3.45	1.99	0.73	–2.72	0.68	5.07
Y@B_12_N_12_	4.41	2.40	1.01	–3.41	0.50	5.77
Y@B_12_N_12_–H_2_	6.18	2.75	1.71	–4.46	0.29	5.81

### 3.5 Electrostatic Potential Analysis

In Figure 6 presents the Molecular Electrostatic Potential (MEP) for the optimized Y@b_64_ and Y@b_64_–H_2_ structures. This analysis demonstrates the variations in the electronic density of the nanocage after modification with Y and adsorption of hydrogen gas, revealing charge distributions as well as the electrophilic and nucleophilic regions of the molecules.^68^

**Figure 6:**
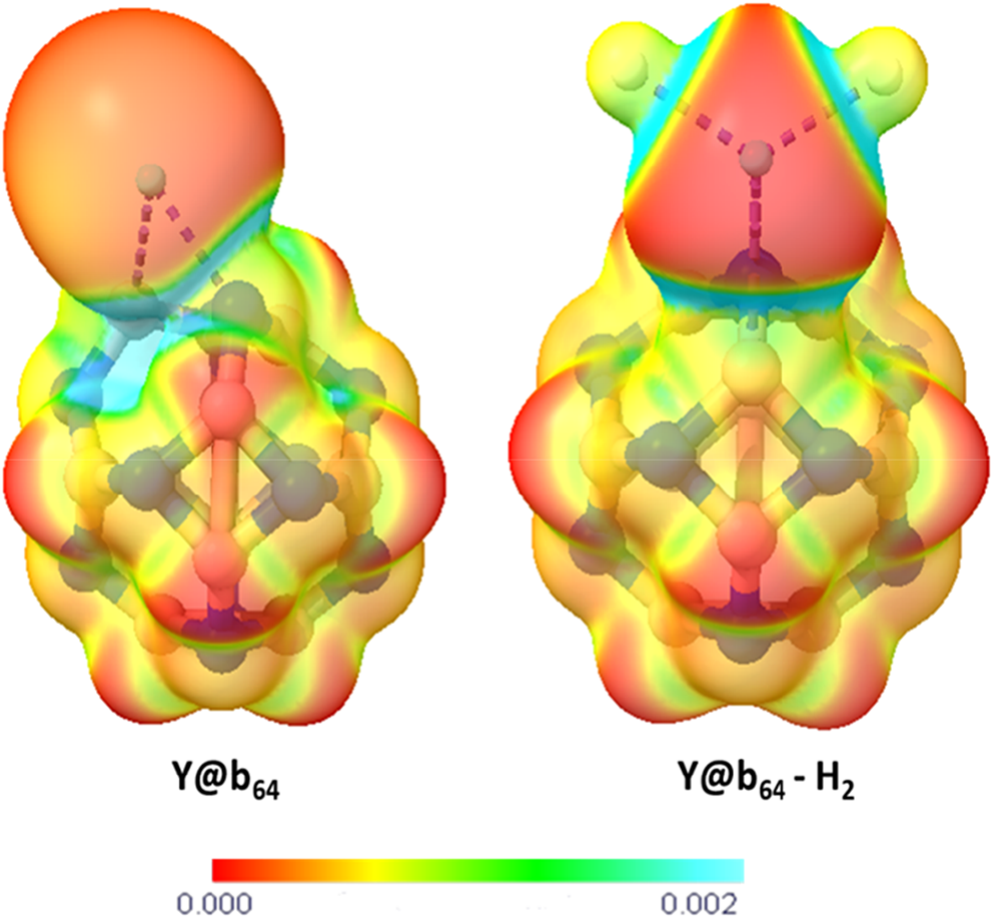
Molecular electrostatic potential (MEP) map of the Y@b_64_ nanocage and the Y@b_64_–H_2_ adsorption system.

The MEP is directly associated with physicochemical properties, such as partial charges, dipole moment, and chemical reactivity. In the analyzed systems, the MEP maps show distinct regions: red and yellow indicate negative charges, corresponding to nucleophilic sites; blue represents positive charges, characterizing electrophilic sites; and light green corresponds to neutral areas.^69,70^ According to Janjua,^71^ in the pristine B_12_N_12_ nanocage, the boron atoms exhibit a positive electrostatic potential (blue regions), whereas the nitrogen atoms display a negative potential (yellow regions). The B_12_N_12_ structure is highly symmetrical and presents a homogeneous charge distribution, which explains its zero dipole moment. Moreover, it was found that H_2_ adsorption on B_12_N_12_ does not induce significant charge redistribution, indicating a weak interaction between the gas and the nanocage.

In this study, the Y@b_64_ decorated structure shows a clear modification in the charge distribution pattern, with the region associated with the yttrium atom exhibiting a nucleophilic character (red regions). After H_2_ adsorption, electrophilic regions emerge along the Y–H bonds, as well as intermediate neutral areas, revealing a stronger interaction between the gas and the modified surface, compared to the interaction of B_12_N_12_ with H_2_.

### 3.6 Thermodynamic Stability Study

Molecular dynamics (MD) analysis was performed in the 0–1000 ps time range, a methodology similar to that employed by De Sousa Sousa et al.^72^ It is noteworthy that this time interval is sufficient to observe possible configurational changes. The stability of the Y@b_64_ nanocage was evaluated before and after the interaction with H_2_, as shown in Figure 7. Previous results by Kundu and Chakraborty^23^ demonstrated that yttrium-doped triazine structures maintain their integrity at high temperatures (420 K), allowing the stable adsorption of up to seven H_2_ molecules per Y atom at 300 K. Similarly, the results obtained for Y@b_64_–H_2_ show that the gas remains adsorbed on the cage surface, presenting only small energetic variations, attributed to geometric adjustments and H_2_ rotations. This stability throughout the simulation confirms the potential of the Y@b_64_ nanocage as a gas sensor, corroborating the chemical interaction data (*E*_ads_ = −0.96 eV) and electronic sensitivity (Δ*E*_gap_ = 74.94%).

**Figure 7:**
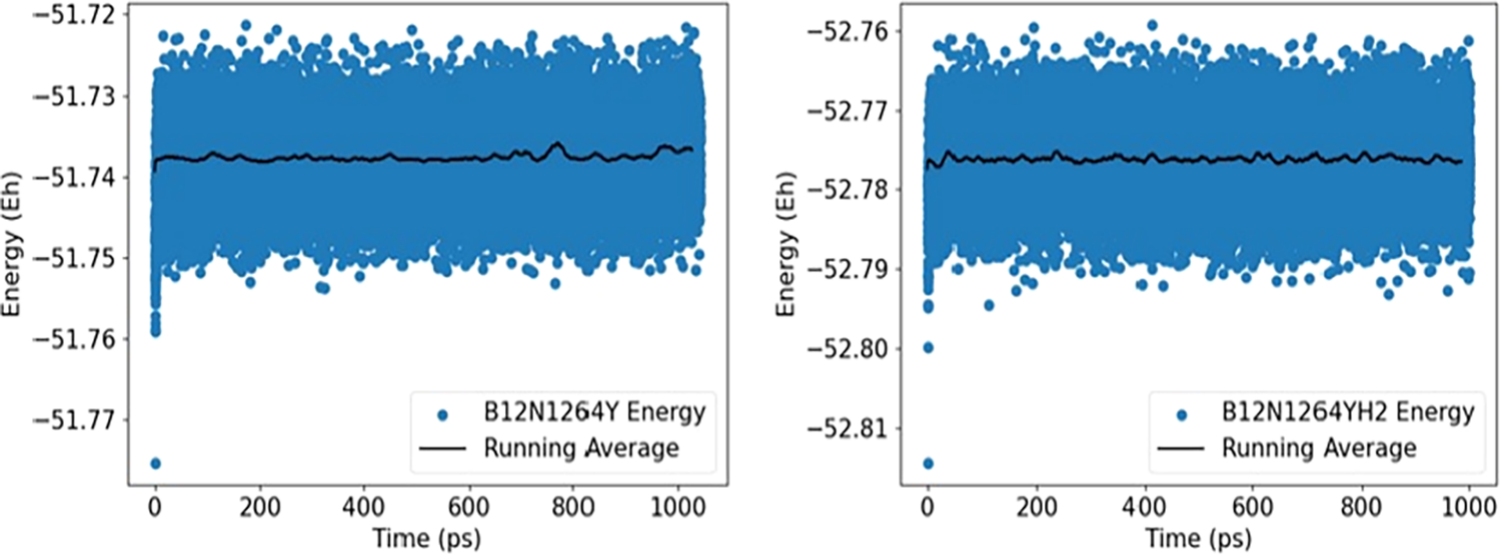
Quantum molecular dynamics trajectory for the Y@b_64_ nanocage before and after interaction with H_2_ gas.

### 3.7 Optical Analysis

The absence of negative frequencies in the spectra confirms the stability of the analyzed geometries (for a more detailed analysis, all frequency values before and after interaction with the H_2_ molecule are presented in Table S2, and the corresponding IR spectra before and after H_2_ adsorption are shown in Figures S2 and S3). The vibrational modes located at approximately 706 and 1632 cm^–1^ correspond to the B–N stretching and B–N bending modes, respectively.^73^ After the adsorption of the H_2_ molecule on the surface of the Y@b_64_ nanocage, the appearance of a peak at 1619 cm^–1^ is observed, which is characteristic of the Y–H interaction.^74^

UV–vis spectroscopy is widely used to monitor adsorption processes. Nair et al.,^75^ for example, applied this technique to monitor the adsorption of 5FU on PLGA. In Figure 8 shows that the Y@b_64_ nanocage exhibits three absorption peaks. The first occurs at 235.8 nm, associated with an excitation energy of *E* = 5.2, with a predominant electronic contribution from the H­(α) → L­(α) transition (49%). The second peak is observed at 314.6 nm, corresponding to *E* = 3.8, resulting from the HOMO → LUMO transitions [H­(α) → L­(α) (27%) and H–1­(α) → L+1­(α) (25%)]. The third peak appears at 453.7 nm, associated with *E* = 2.5, with a greater contribution from the H­(α) → L­(α) transition (72%). After adsorption of the H_2_ molecule, the Y@b_64_–H_2_ system presented a maximum absorbance peak at λ_max_ = 219.2 nm, corresponding to an excitation energy of *E* = 5.6, with contributions from the transitions [H­(β) → L­(β) (26%) and H–1­(β) → L+1­(β) (20%)]. Furthermore, a peak was identified at λ_min_ = 339.0 nm, associated with E = 3.5, with electronic transition [H­(α) → L­(α) (83%)].^76^ Wavelengths (λ_max_), oscillator intensities (*f*), energies (*E*), and main electronic transitions associated with the absorption peaks of the Y@b_64_ and Y@b_64_-H_2_ systems are presented in Table S3. The spectral shift thus provides a promising experimental signature for in situ validation of gas adsorption via UV–vis.

**Figure 8:**
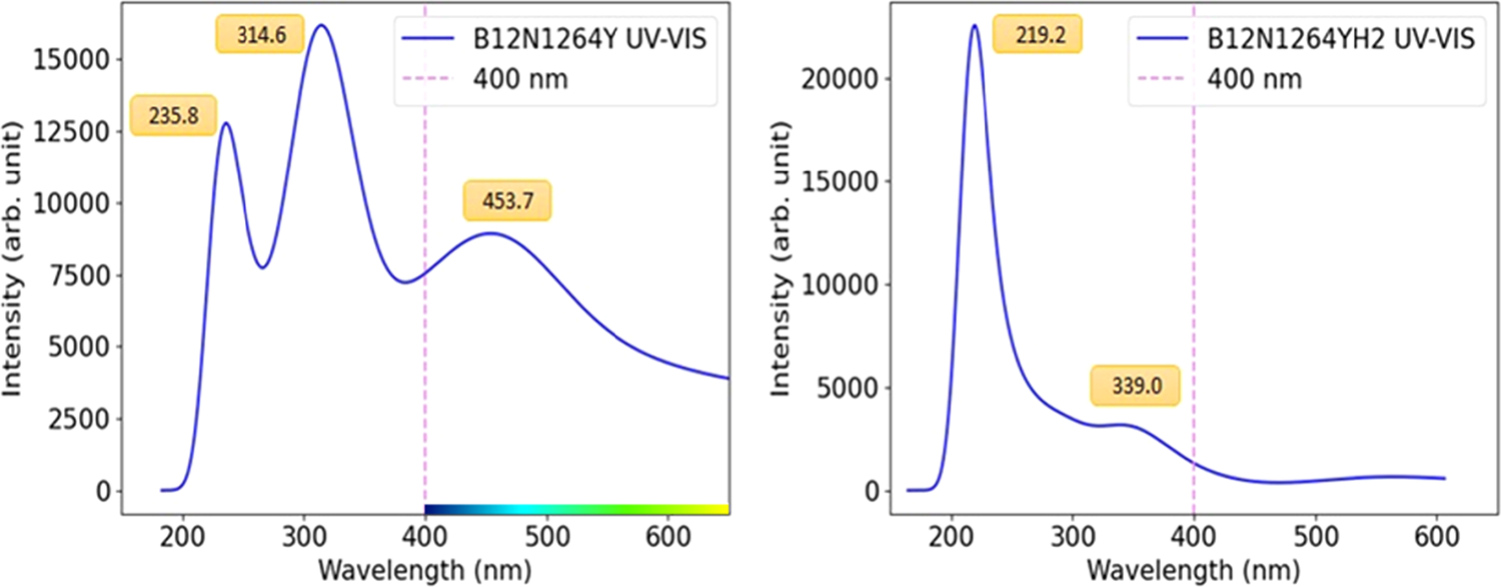
UV–vis spectrum of the Y@b_64_ nanocage before and after H_2_ gas adsorption.

The changes in the UV–vis spectrum of the Y@b_64_–H_2_ system occur due to the absorption of the H_2_ molecule by the Y atom. The results indicate that the yttrium-decorated nanocage Y@b_64_ exhibits optical sensitivity to H_2_ gas, suggesting that this material possesses characteristics suitable for application in optical hydrogen gas detection.

### 3.8 Study of Adsorption of Interfering Gases

The selectivity of the Y@b_64_ nanocage for H_2_ was evaluated relative to some interfering gases commonly used in previous experimental studies reported in the literature (CH_4_, CO, H_2_S, and N_2_)^24,25,77,78^ (see Figure 9). The adsorption distances showed that H_2_ interacts more strongly (*d* = 1.98 Å) than the interfering gases, which presented larger distances (2.39 to 3.56 Å) and weak interactions. The NPA charge analysis of the interfering gases showed that the charge variation on the Y atom was 1.782, whereas for the H_2_ molecule it was −1.147, indicating a significantly greater charge transfer from the Y@b_64_ nanocage to H_2_ compared with the gases CO, CH_4_, H_2_S, and N_2_. H_2_ adsorption (*E*_ads_ = −0.96 eV) characterizes chemisorption, with an adequate recovery time (τ = 166.8 s), while the interferents present very short recovery times. These results confirm the high selectivity and efficiency of Y@b_64_ in the detection of hydrogen gas. However, as pointed out by Kaewmaraya et al.,^38^ we need to treat the calculated binding energy with caution because this value is based on the single molecule adsorption which does not take into, for example, the factor of gas concentration feeding to the sensor.

**Figure 9:**
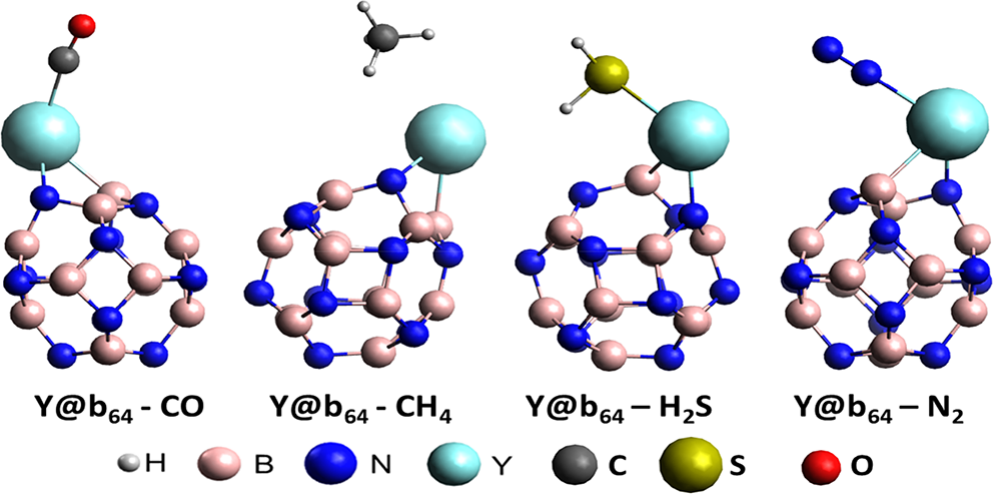
Optimized structures of the adsorption of Y@b_64_ nanocage with interfering gases CO, CH_4_, H_2_S, and N_2_.

The Δ*E*_gap_ results show that the Y@b_64_ nanocage presents greater sensitivity to hydrogen gas compared to the other gases evaluated (Δ*E*_gap_ = 74.94%), indicating that the system preferentially detects H_2_ (data presented in Table 5). The variation in the free energy of adsorption (Δ*G*_ads_) confirms that the adsorption processes are spontaneous for all gases studied, except for methane (Δ*G*_ads_ = +0.32 eV), characterized as nonspontaneous. Selectivity was analyzed by calculating the sensor response (*S*) and the selectivity coefficient (κ), according to eqs 17 and 18, parameters that express the efficiency of the system in the selective detection of H_2_ compared to the tested atmospheric gases. Greater selectivity was observed for the H_2_/CH_4_ pair, reinforcing the lower detection of methane and confirming the high sensitivity of Y@b_64_ to hydrogen gas.

**Table 5:** Values of Gap HOMO-LUMO (*E*_gap_), Cage/Gas Distance (*d*), NPA Charge on the Y Atom (*Q*_Metal_) and NPA Charges on the Atoms of Interfering Gases (*Q*_gas_), Adsorption Energy (*E*_ads_), Electronic Sensitivity (Δ*E*_gap_), Free Energy of Adsorption (Δ*G*_ads_), Recovery Time (τ), the Sensitivity (*S*) and Selectivity Coefficient (κ) Calculated for the Interaction of CO, CH_4_, H_2_S, and N_2_ Gases with the Y@b_64_ Cage

system	*E*_gap_ (eV)	*d* (Å)	*Q*_Metal_ (|e|)	*Q*_gas_ (|e|)	*E*_ads_ (eV)	Δ*E*_gap_ (%)	Δ*G*_ads_ (eV)	τ	*S*	κ
Y@b_64_–H_2_	5.12	1.98	1.782	–1.147	–0.96	74.94	–0.40	166.80 s	3.19 × 10^18^	
Y@b_64_–CO	1.77	2.39	1.413	0.002	–0.62	27.12	–0.03	299.87 μs	6.33 × 10^9^	5.04 × 10^8^
Y@b_64_–CH_4_	3.01	3.56	1.170	–0.295	–0.06	2.76	+0.32	103.25 fs	3.74	8.53 × 10^17^
Y@b_64_–H_2_S	2.32	2.83	1.110	0.052	–0.65	4.65	–0.04	963.55 μs	1.43 × 10^5^	2.23 × 10^13^
Y@b_64_–N_2_	1.75	2.39	1.258	–0.177	–0.33	28.00	–0.07	3.77 ns	9.33 × 10^9^	3.42 × 10^8^

Table 6 shows the performance of different nanomaterials in H_2_ detection reported in the literature,^11,79–88^ whose adsorption energies range from −0.072 to −1.830 eV. Values in the range −0.3 eV < *E*_ads_ < −1.0 eV are considered ideal for sensor applications.^37,38,57^ Pure B_12_N_12_ presented *E*_ads_ = −0.072 eV, characterizing physisorption, a behavior similar to other nanomaterials, such as AlP-doped graphene (*E*_ads_ = −0.218 eV), NiN_4_S-doped SWCNT (*E*_ads_ = −0.082 eV), and C_16_Mg_8_O_8_ nanocage (*E*_ads_ = −0.170/–0.114 eV). It is noteworthy that the rGO-ZnO–Ag-Pd film and Al_12_C_12_ nanocage systems, despite presenting adequate adsorption energies and recovery times, as well as the Y@b_64_–H_2_ system (*E*_ads_ = −0.96 eV and τ = 166.8 s), proposed in this work. The Y@b_64_ nanocage, in comparison to the systems presented in Table 6, presented the highest electronic sensitivity to H_2_ (Δ*E*_gap_ = 74.94%), in addition to being selective to CH_4_, CO, H_2_S and N_2_ gases, positioning the Y@b_64_ nanocage as a promising material for application in selective H_2_ gas sensors.

**Table 6:** Comparison of Sensing Materials for the Detection of Hydrogen Gas (H_2_)

sensor	functional	*E*_ads_ (eV)	Δ*E*_gap_ (%)	τ (s)	other gases	refs
B_12_N_12_ nanocage	B3LYP-D4	–0.072	5 × 10^–3^ ^a^		CO_2_	Choir et al.^11^
C_16_Mg_8_O_8_ nanocage	M06-D3	–0.170	0.54^a^		N_2_	Ghamsari et al.^79^
	B97D	–0.114	3.45^a^			
rGO-ZnO–Ag-Pd film	GGA-PBE	–0.720	59.00^c^	14.00		Pal et al.^80^
W-doped graphene	GGA-PBE-D2	–1.035	24.85^b^	2.44 × 10^5^	NH_3_, CH_4_, CO, SO_2_ and H_2_S	Yang et al.^81^
AlP-decorated graphene	M06–2X	–0.218	4.68^b^	4.65 × 10^–9^		Zakeri et al.^82^
Pt-doped g-C_3_N_4_	GGA-PBE	–1.830	26.19^a^		CH_4_ and CO_2_	Luo et al.^83^
Pt-ZnONT	B3LYP-D3	–1.360	4.19^a^	1.35 × 10^–9^ ^d^		Li and Asad^84^
Pt-decorated WS_2_	GGA-PBE-D	–1.300	27.00^a^	1.85^e^	CO, H_2_S, NH_3_, and CH_4_	Li et al.^85^
Pt-doped MoTe_2_	GGA-PBE-D	–1.791	12.41	1.32 × 10^6^ ^f^	CO, C_2_H_4_, and C_2_H_2_	Jiang et al.^86^
NiN_4_S-doped SWCNT	ωB97XD	–0.082	50.94^b^	2.20 × 10^–11^		Imeni et al.^87^
Al_12_C_12_ nanocage	B3LYP	H_2_–Al = −0.550	0.21^c^	1.97 × 10^–3^	CH_4_, CO, NO, and NH_3_	Huang et al.^88^
		H_2_–C = −0.560	0.21^c^	2.90 × 10^–3^		
Y@b_64_	B3LYP-D3	–0.960	74.94	166.80	CH_4_, CO, H_2_S, and N_2_	this work

a Value calculated from *E*_gap_ data.

b Value found in the article.

c Value calculated by the equation *S* = [(*R* – *R*_0_)/*R*_0_] × 100%.

d Value calculated at 598 K.

e Value calculated at 538 K.

f Value calculated at 498 K.

The comparison among different theoretical studies of H_2_ sensors highlights the central role of computational modeling in identifying materials with superior performance in terms of sensitivity, selectivity, and stability. The detailed evaluation of electronic, structural, and adsorption properties not only guides the rational selection of promising candidates but also reduces experimental costs and directs the development of more efficient devices. These advances hold potential for application in leak-monitoring systems, safety devices for hydrogen production, and technologies associated with fuel cells. Thus, theoretical results not only deepen the understanding of detection mechanisms but also strengthen the integration between computational research and the implementation of technological solutions, driving the transition toward the growing hydrogen economy.

## 4 Conclusions

In this study, density functional theory (DFT) and time-dependent DFT (TD-DFT) calculations revealed that yttrium modification of B_12_N_12_ nanocages significantly enhances H_2_ adsorption performance. Geometric, electronic, energetic and optical properties were analyzed, and the results presented that while the pristine B_12_N_12_ nanocage exhibited weak physisorption (*E*_ads_ = −0.04 eV; Δ*G*_ads_ = +0.25 eV), all Y-modified configurations displayed spontaneous adsorption (Δ*G*_ads_ < 0) with markedly increased interaction energies. Among the studied systems, the Y@b_64_–H_2_ configuration exhibited chemisorption (*E*_ads_ = −0.96 eV) with adequate recovery time (τ = 166.8 s), substantial electronic sensitivity (Δ*E*_gap_ = 74.94%), structural stability under molecular dynamics simulations, and a detectable optical response in the UV–Vis region. Furthermore, it demonstrated superior selectivity against common interfering gases (CH_4_, CO, H_2_S, and N_2_), highlighting its potential as a dual-mode electronic and optical H_2_ sensor. These results position Y@b_64_ as a highly promising candidate for hydrogen monitoring and industrial safety applications. Experimental validation will be essential to confirm these theoretical predictions under practical conditions, particularly in industrial safety and hydrogen monitoring environments. Future directions include investigating the integration of this material into real sensing platforms, as well as assessing its performance in miniaturized devices operating under variable environmental conditions, targeting applications in intelligent detection networks and in the emerging infrastructure of the hydrogen economy.

## Supplementary Material



## Notes

DOS of hydrogen gas adsorption on pure B_12_N_12_, YB_11_N_12_, B_12_N_11_Y, Y@b_64_, Y@b_66_ and Y@B_12_N_12_ nanocages (Figure S1); IR spectra of the of the B_12_N_12_ nanocage and the yttrium metal-modified nanocages: doped (YB_11_N_12_ and B_12_N_11_Y), decorated (Y@b_64_ and Y@b_66_), and encapsulated (Y@B_12_N_12_) (Figure S2); IR spectra of the hydrogen gas (H_2_) adsorption on the surfaces of pure and Y-modified B_12_N_12_ nanocages (Figure S3); Multiplicity, sum spin population, Mulliken Spin Population, Electron Population, *S* ^2^, deviation, effective magnetic moment (μ_ef_), and magnetism for all systems studies (Table S1); Vibrational frequencies (cm^–1^) for all systems studies (Table S2); Wavelengths (λ_max_), oscillator intensities (*f*), energies (*E*), and main electronic transitions associated with the absorption peaks of the Y@b_64_ and Y@b_64_-H_2_ systems (Table S3) (PDF)
