# Supplementary material for: Yttrium-Modified B12N12 Nanocages for High-Performance H2 Sensing: Insights from DFT Calculations on Sensitivity, Selectivity, and Recovery
Source: ACS Omega. 2026 Jan 21;11(4):6421–33. doi: 10.1021/acsomega.5c11273 (PMC12878730; doi:10.1021/acsomega.5c11273)
Supplement: Supplementary file 1 [file ao5c11273_si_001.pdf]

Yttrium-Modified $B_{12}N_{12}$ Nanocages for High-Performance H_2 Sensing: Insights from DFT Calculations on Sensitivity, Selectivity, and Recovery

Wellington da Conceição Lobato do Nascimento[1], Natanael de Sousa Sousa[1], Adilson Luís Pereira Silva[2*], Adeilton Pereira Maciel[3]

Affiliations

[1] *Universidade Federal do Maranhão, 65080-805, São Luís, MA, Brazil.*
[2] *Universidade Estadual do Maranhão, 65055-310, São Luís, MA, Brazil.*
[3] *Universidade Federal do Rio Grande do Norte, 59078-970, Natal, RN, Brazil.*
* Corresponding Author. E-mail address: adlpsilva@gmail.com

Figure S1 – DOS of hydrogen gas adsorption on pure $B_{12}N_{12}$, $YB_{11}N_{12}$, $B_{12}N_{11}Y$, Y@b_{64}, Y@b_{66} and Y@$B_{12}N_{12}$ nanocages.

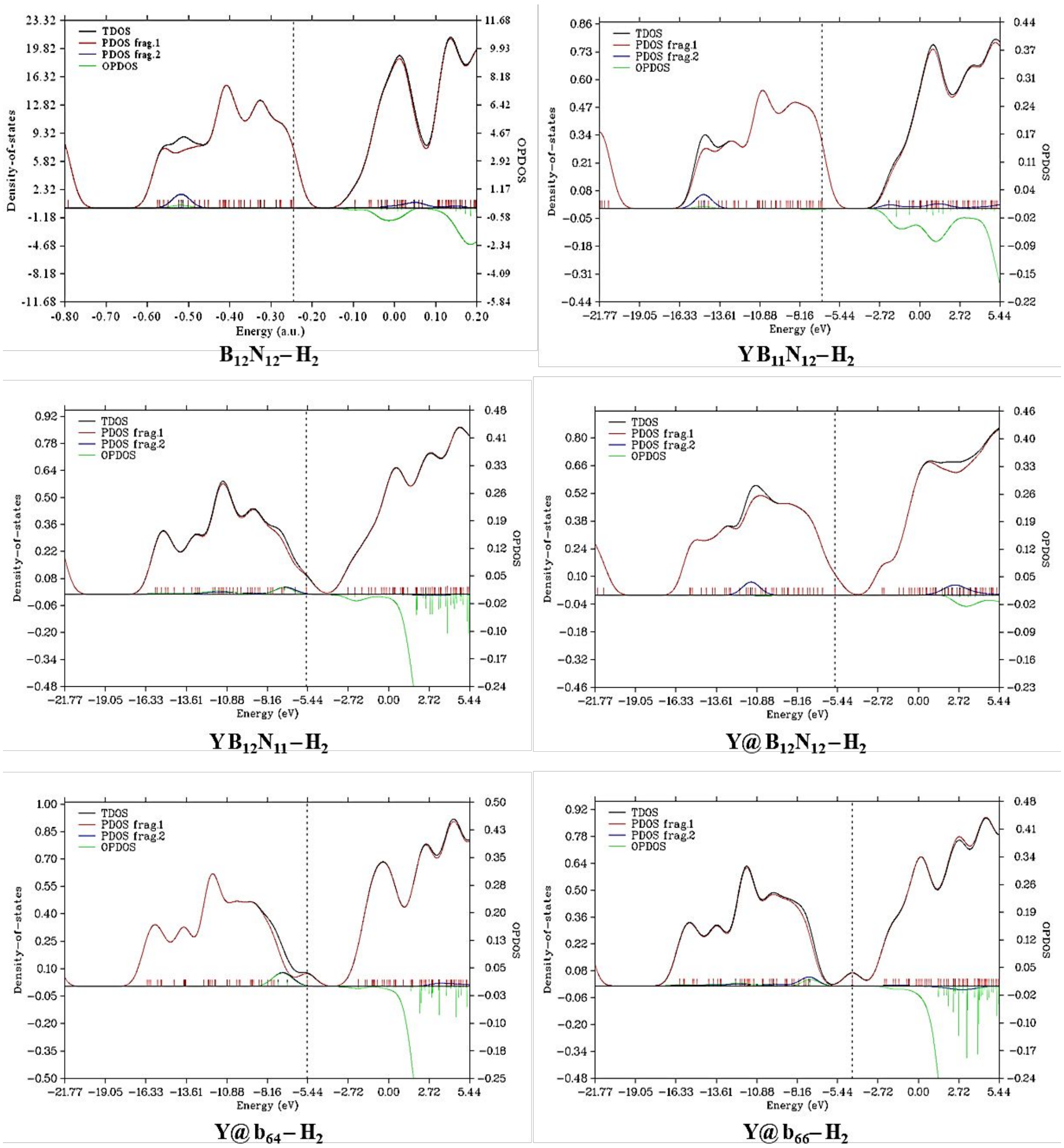

Figure S2 – IR spectra of the of the $B_{12}N_{12}$ nanocage and the yttrium metal-modified nanocages: doped ($YB_{11}N_{12}$ and $B_{12}N_{11}Y$), decorated (Y@b_{64} and Y@b_{66}), and encapsulated (Y@$B_{12}N_{12}$).

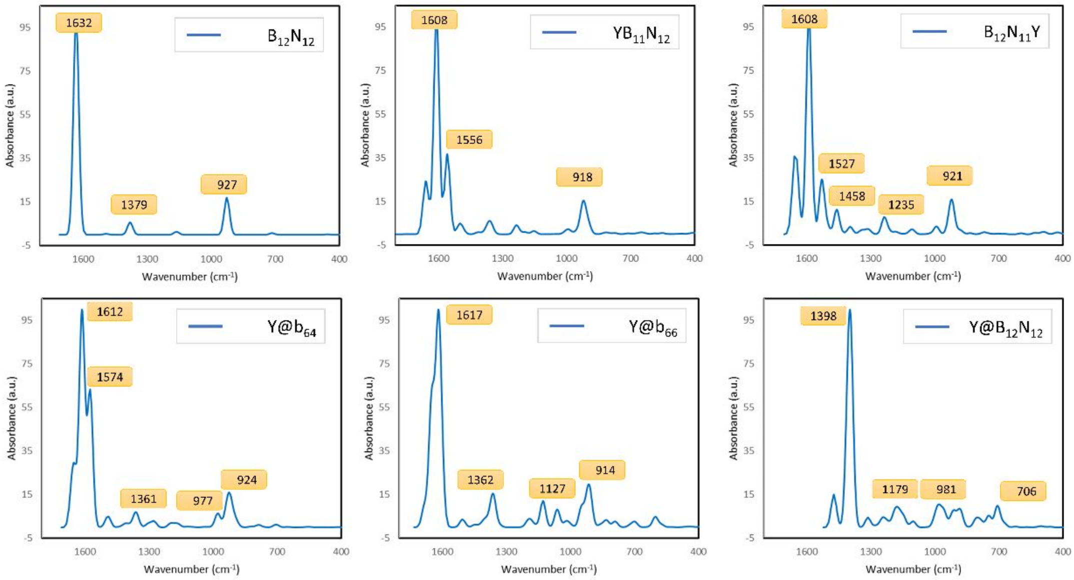

Figure S3 – IR spectra of hydrogen gas (H_2) adsorption on the surfaces of pure and Y-modified $B_{12}N_{12}$ nanocages.

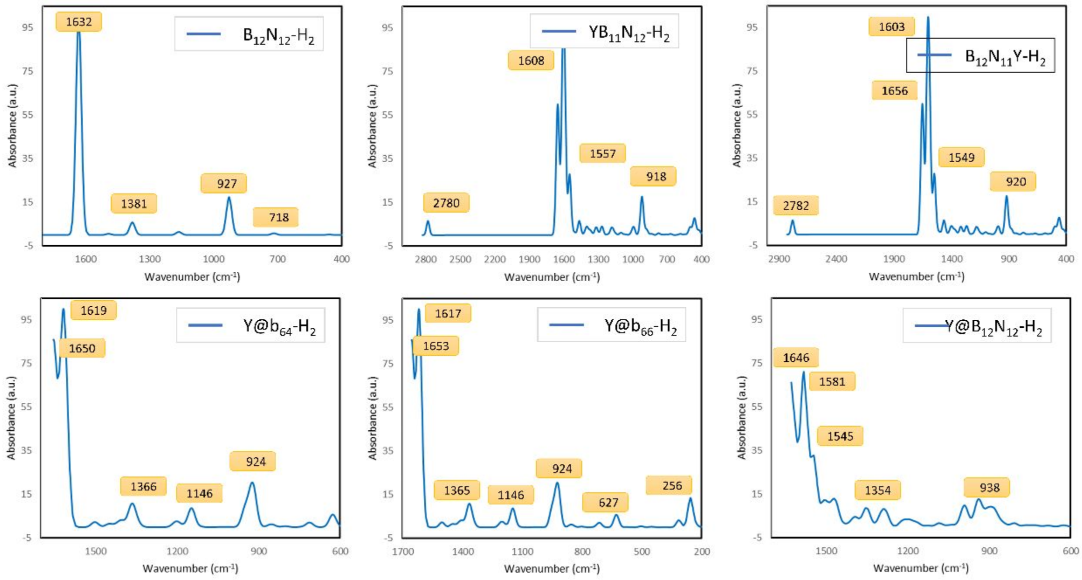

Table S1 – Multiplicity, sum spin population, Mulliken Spin Population, Electron Population, S^2, deviation, effective magnetic moment (μ_{ef}), and magnetism for all systems studies.

Systems	Multiplicity	Sum Spin Population	Mulliken Spin Population			Electron Population	S^2	Deviation	μ_{ef}	Magnetism
			Y	H	H	Y				
$B_{12}N_{12}$	1	0					0.000	0.000	0.000	No
$YB_{11}N_{12}$	1	0				36.9	0.000	0.000	0.000	No
$B_{12}N_{11}Y$	1	0				37.7	0.000	0.000	0.000	No
$Y@b_{64}$	2	1	0.939			37.8	0.751	0.001	1.733	Yes
$Y@b_{66}$	2	1	0.840			38	0.760	0.010	1.744	Yes
$Y@B_{12}N_{12}$	2	1	0.008			38.4	1.749	0.999	2.645	Yes
$B_{12}N_{12}H_2$	1	0					0.000	0.000	0.000	No
$YB_{11}N_{12}$-H_2	1	0				36.9	0.000	0.000	0.000	No
$B_{12}N_{11}Y$-H_2	1	0				37.4	0.000	0.000	0.000	No
$Y@b_{64}$-H_2	2	1	0.282	0.0079	-0.0015	37.2	0.752	0.002	1.734	Yes
$Y@b_{66}$-H_2	2	1	1.070	0.006	0.01	37.6	0.751	0.001	1.733	Yes
$Y@B_{12}N_{12}H_2$	2	1	0.170	0.98	1.01	37.2	0.764	0.014	1.748	Yes

Table S2 – Vibrational frequencies (cm^{-1}) for all systems studies.

	**$B_{12}N_{12}$**	
322.56	688.99	1011.08
322.67	748.65	1011.25
322.71	748.75	1050.11
373.33	748.76	1050.12
373.35	750.05	1176.32
396.74	750.10	1176.45
396.78	798.53	1176.60
396.83	806.09	1199.68
437.93	806.17	1199.76
437.96	806.18	1199.81
505.05	806.42	1243.28
505.05	839.20	1243.35
505.08	839.46	1243.42
560.11	839.52	1297.06
560.30	879.09	1297.14
560.30	902.17	1297.31
622.42	907.98	1309.37
622.89	908.07	1419.09
622.94	908.35	1419.13
668.55	914.72	1419.20
688.75	914.72	1444.89
688.91	1010.93	1444.89
	**$B_{12}N_{12}$-H_2**	
42.36	668.39	1011.14
55.95	688.10	1011.45
114.13	689.05	1011.66
147.60	689.42	1050.18
277.11	748.22	1050.54
322.97	748.38	1176.56
324.11	748.91	1176.87
324.43	750.39	1177.31
374.79	751.77	1200.36
374.87	798.83	1200.90
398.14	805.83	1201.15
398.58	806.80	1244.17
398.91	807.05	1244.49
440.04	807.42	1244.69
440.35	838.14	1296.69
505.66	839.31	1297.30
506.19	839.62	1298.06
506.28	879.34	1309.42
561.25	901.79	1418.76
563.08	908.31	1419.12
563.41	908.38	1419.47
621.35	909.43	1444.56
624.89	915.45	1445.21
625.18	915.70	4374.21

$YB_{11}N_{12}$		
162.80	641.09	947.29
192.45	671.77	1001.14
264.04	690.93	1002.87
271.98	698.94	1037.49
311.60	712.98	1053.59
319.57	726.20	1071.03
349.49	736.89	1153.07
370.07	749.65	1170.19
370.38	777.39	1178.71
399.50	780.76	1181.87
402.70	797.68	1196.23
426.21	803.02	1216.32
449.41	804.33	1228.32
479.88	814.40	1230.57
505.46	823.05	1291.58
545.19	844.88	1292.26
545.96	863.23	1304.65
563.37	874.76	1353.77
573.16	880.99	1398.63
592.95	893.97	1398.76
617.31	912.17	1432.65
621.44	918.43	1442.55

$YB_{11}N_{12}$-H_2		
39.86	593.13	917.97
49.57	617.56	946.92
79.36	622.33	1000.70
159.42	641.18	1002.77
185.26	671.80	1037.58
263.48	691.02	1053.86
273.63	699.27	1073.01
281.54	713.07	1151.53
311.52	725.73	1170.20
319.79	736.94	1178.75
350.71	750.24	1182.82
369.82	777.71	1196.81
370.80	781.18	1216.21
399.80	798.26	1228.60
403.34	803.03	1230.82
426.36	804.17	1291.99
449.76	814.57	1292.39
479.62	823.72	1304.89
505.33	844.79	1354.29
511.67	863.32	1398.14
545.49	874.00	1398.77
546.32	880.91	1432.33
563.55	893.77	1443.41
573.86	912.68	4327.23

$YB_{12}N_{11}$		
137.10	613.04	959.98
150.36	644.34	971.64
205.35	667.38	999.35
207.22	685.61	1027.98
210.89	688.92	1055.97
321.23	724.02	1075.10
331.70	725.21	1126.64
366.26	734.74	1141.00
367.91	763.37	1164.51
384.25	770.40	1176.59
418.38	779.11	1178.51
423.81	798.17	1213.15
435.38	800.89	1214.34
438.43	810.06	1244.55
454.52	813.65	1268.49
462.17	862.34	1305.52
512.51	863.28	1307.71
516.70	886.02	1329.21
527.96	891.96	1380.29
541.00	899.99	1383.27
584.07	912.76	1430.38
591.93	923.90	1440.87

$YB_{12}N_{11}$-H_2		
101.15	615.96	1000.81
143.61	642.50	1020.70
161.84	673.54	1031.78
190.01	685.89	1060.79
223.64	692.36	1102.79
303.61	723.96	1120.95
325.21	731.92	1140.32
332.34	736.74	1147.59
364.05	763.32	1183.97
367.91	767.40	1195.22
379.20	781.21	1203.59
402.49	798.43	1217.79
412.38	800.57	1232.10
419.03	809.06	1264.42
432.69	814.44	1275.11
439.16	859.96	1307.82
453.78	863.20	1316.43
476.63	879.56	1347.35
508.80	888.66	1384.14
511.87	899.59	1399.23
531.44	912.52	1433.82
540.74	923.47	1437.87
583.01	936.68	1444.21
584.77	959.89	2419.53

Y@b$_{64}$		
71.27	659.33	949.61
148.06	664.11	972.05
165.57	683.42	1007.12
206.62	688.62	1020.15
285.30	710.49	1041.88
330.70	718.11	1109.51
346.01	748.09	1132.83
359.57	752.28	1172.14
381.70	765.47	1182.64
386.97	777.10	1184.71
402.95	788.21	1188.46
425.46	798.14	1204.46
448.00	805.31	1224.08
475.76	816.87	1233.07
484.76	835.29	1249.78
501.54	850.19	1294.42
518.61	866.20	1302.07
544.64	878.51	1315.54
564.22	899.08	1368.76
571.30	901.63	1400.14
600.43	909.14	1405.01
610.05	915.51	1434.77
619.09	918.18	1444.21
**Y@b$_{64}$-H$_2$**		
46.32	622.37	968.91
89.62	645.46	996.88
154.94	676.46	1015.48
199.75	685.64	1037.93
222.78	694.06	1041.22
273.12	704.15	1052.12
307.45	735.38	1166.72
328.47	738.86	1183.97
336.71	748.98	1191.41
367.91	754.54	1193.80
379.34	787.36	1195.63
388.75	796.29	1221.43
396.70	798.49	1236.91
434.14	801.84	1244.08
438.09	810.74	1261.56
443.49	812.47	1301.54
481.68	827.10	1307.12
500.07	835.14	1308.73
504.49	876.34	1399.01
545.20	889.12	1408.48
553.66	898.34	1428.19
555.02	901.96	1437.84
559.38	909.35	1439.25
615.02	915.67	1456.86
620.51	918.47	1488.24

**Y@b$_{66}$**		
60.58	662.92	922.57
132.72	675.25	980.61
172.46	683.63	1010.50
261.93	687.77	1029.40
291.16	720.80	1039.50
327.02	722.31	1045.95
337.59	745.11	1157.69
374.91	749.40	1181.92
376.50	768.15	1186.18
393.50	787.31	1191.80
405.06	790.57	1197.67
424.97	795.03	1215.67
434.45	801.49	1230.46
456.57	802.40	1243.65
487.48	823.94	1258.22
498.19	824.22	1299.12
523.51	869.46	1307.52
542.00	877.05	1310.68
552.00	885.67	1394.21
552.22	892.45	1407.11
606.37	902.44	1431.56
611.76	912.00	1437.40
626.34	914.99	1463.08
**Y@b$_{66}$-H$_2$**		
50.31	665.03	1006.49
103.42	677.54	1020.78
167.33	688.68	1031.74
230.19	691.39	1058.42
278.76	724.67	1123.43
300.77	733.66	1128.79
322.13	752.29	1166.73
335.12	760.95	1182.13
356.20	774.84	1200.37
381.75	781.07	1205.25
385.21	788.07	1214.74
393.88	803.12	1238.99
408.79	807.23	1251.34
437.40	813.10	1260.62
449.51	837.21	1286.63
486.35	847.83	1306.81
496.88	867.04	1315.70
501.99	889.81	1341.36
526.20	906.37	1384.55
553.57	911.84	1412.05
562.21	914.86	1418.13
578.46	916.58	1438.60
613.55	918.92	1451.54
627.76	932.51	1487.37
635.50	971.93	2131.06

**Y@$B_{12}N_{12}$**		
265.46	648.47	859.52
268.63	651.93	870.55
307.88	679.21	902.52
398.20	683.58	957.48
398.73	686.33	958.60
413.28	702.48	960.61
415.67	709.06	996.50
425.63	729.02	999.46
435.70	733.02	1018.24
440.12	744.08	1033.80
440.62	763.03	1035.84
453.85	768.99	1064.31
454.63	772.55	1082.85
462.08	783.84	1084.63
495.43	791.30	1110.79
495.90	792.38	1142.82
541.04	795.28	1143.45
541.94	797.11	1208.51
542.28	805.61	1212.21
587.70	832.21	1216.01
610.82	833.58	1225.84
614.38	852.28	1281.57
616.72	856.15	1282.52
**Y@$B_{12}N_{12}$-H_2**		
11.45	548.98	925.31
35.02	584.61	942.58
126.77	593.13	978.29
131.52	604.89	1012.79
166.76	629.62	1033.38
195.17	643.99	1046.12
228.56	665.97	1060.90
257.04	701.09	1111.50
260.08	707.61	1117.99
268.72	726.53	1129.66
302.67	735.33	1169.04
321.02	750.85	1179.71
346.33	766.50	1208.72
359.46	771.00	1222.14
362.41	785.82	1245.83
379.28	795.94	1272.47
396.00	810.99	1283.16
417.97	816.19	1292.42
427.60	828.72	1310.83
446.35	852.77	1343.80
459.48	860.31	1374.94
478.03	864.31	1407.01
482.35	872.47	1431.39
518.39	899.74	1467.15
539.37	913.33	4371.54

Table S3 – Wavelengths (λ_{max}), oscillator intensities (f), energies (E), and main electronic transitions associated with the absorption peaks of the Y@b_{64} and Y@b_{64}-H_2 systems.

System	λ_{max} (nm)	f	E (eV)	Transition
Y@b_{64}	235.8	0.047	5.2	H(α) → L(α) (49%)
				H(β) → L(β) (37%)
	314.6	0.15	3.8	H(α) → L(α) (27%)
				H-1(α) → L+1(α) (25%)
	453.7	0.062	2.5	H(α) → L(α) (72%)
				H-1(α) →L+1(α) (22%)
Y@b_{64}-H_2	219.2	0.083	5.6	H(β) → L(β) (26%)
				H-1(β) → L+1(β) (20%)
				H(α) → L(α) (19%)
	339	0.042	3.5	H(α) → L(α) (83%)
